# Fertilization competence of the egg-coating envelope is regulated by direct interaction of dicalcin and gp41, the *Xenopus laevis* ZP3

**DOI:** 10.1038/srep12672

**Published:** 2015-08-05

**Authors:** Naofumi Miwa, Motoyuki Ogawa, Mayu Hanaue, Ken Takamatsu

**Affiliations:** 1 Department of Physiology, School of Medicine, Toho University, Ohmori-nishi 5-21-16, Ohta-ku, Tokyo 143-8540, Japan; 2 Department of Anatomy, School of Medicine, Kitasato University, Kitasato 1-15-1, Minami-ku, Sagamihara, Kanagawa 228-8555, Japan

## Abstract

Fertilization begins with species-restricted interaction of sperm and the egg-coating envelope, which includes a three-dimensional meshwork of filaments composed of glycoproteins (called ZP proteins). Growing evidence has unveiled the molecular nature of ZP proteins; however, the structural property conferring fertilization competence to the egg-coating envelope remains unknown. Here, we show the molecular mechanism that mediates direct interaction between dicalcin, a novel fertilization-suppressive ZP protein-associated protein, and gp41, a *Xenopus laevis* ortholog of mammalian ZP3, and subsequently demonstrate the structural basis of the envelope for fertilization competence. The interactive regions between dicalcin and gp41 comprised six and nine amino acid residues within dicalcin and twenty-three within gp41. Synthetic peptides corresponding to these regions dramatically affected fertilization: treatment with dicalcin- or gp41-derived peptides decreased or increased fertilization rates, respectively. Prior application of these peptides caused distinct alterations in the *in vivo* lectin-staining pattern of the envelope as well. Transmission electron microscopy analysis revealed that the dicalcin-derived peptide induced the formation of a well-organized meshwork, whereas the gp41-derived peptide caused the formation of a significantly disorganized meshwork. These findings indicated that the fertilization competence of the egg-coating envelope is crucially regulated by the direct interaction between dicalcin and gp41.

In general, mature oocytes of animals are surrounded by an extracellular envelope called either the zona pellucida (ZP) in mammals and vitelline envelope (VE) in nonmammals^1^. This extracellular matrix plays multiple roles in the generation and development of the zygote, including species-selective interaction between gametes, induction of acrosome reaction, prevention of polyspermy by structural modifications following fertilization, and protection of the fragile early embryo from physical damage^2^. The ZP contains a three-dimensional meshwork of filaments formed by polymerization of ZP proteins that are mainly secreted from growing oocytes with posttranslational modification by glycosylation. ZP proteins of the egg-coats comprises three to seven ZP proteins, including ZP1-4 in humans, ZP1-3 in mice, ZP1-ZPX in birds, and gp120, gp69/64, gp41, and gp37 in *Xenopus laevis*^3^,^4^. These proteins associate with other ZP proteins via their conserved motif called the ZP domain, thus creating μm-long filaments that interconnect to form a three-dimensional meshwork^5^. ZP3 has been considered to be the primary sperm-binding target through its presentation of O-glycan at T168 (site 1) and T177 (site 2) in the mouse ZP3, and N-glycan at N82 in the frog ZP3, together with a variable ZP-C subdomain recently implicated in association with different ZP proteins as well as sperm recognition^6^,^7^. On the other hand, the polypeptide moiety of ZP2 is also considered to be important for sperm binding^8^, and it is thought that the particular three-dimensional structure of the ZP may make the structure susceptible to sperm binding^9^. Structural changes in the ZP meshwork have been observed during egg development and postfertilization in a variety of animals^10^,^11^. In *X. laevis* egg, for example, extracellular structures include an outer and inner egg coat called the egg jelly and VE. Of these egg coats, the sperm-impenetrable premature VE contains ZP filament fibers (4–7 nm in diameter), each of which bundles together, giving the appearance of a coarse filter paper with a “tunnel-like” open structure of ~0.04 μm^2^ among ZP-filament lattices^12^. Concomitant with limited proteolysis of gp41 by oviductin, an oviductal protease^13^, within the oviduct, the egg-coating envelope is converted to a sperm-penetrable status, in which the individual fibers of bundles are dispersed uniformly within the envelope and arranged parallel to the egg surface, resulting in the disappearance of large open spaces^14^. Following fertilization, the resulting VE is again converted to the sperm-impenetrable status (fertilization envelope [FE]) to block polyspermy through the sperm-egg fusion-triggered release of the cortical granule contents. The FE appears to be multilayered with fibrous sheets that twist and curl and sometimes merge; in addition, the FE is elevated away from the egg surface, widening the perivitelline space between the envelope and egg surface^14^. Despite these studies, few reports have described the structural diversity of the ZP in mature unfertilized eggs, with the exception of the immunohistochemical examinations showing the heterogeneous lectin-staining patterns of the ZP of fertilization-failed eggs^15^. In particular, no studies have demonstrated the fine structural basis for the fertilization competence of mature ZP, partly owing to the absence of a reagent to clamp the VE in the competent or incompetent status. Therefore, the type of three-dimensional meshwork that facilitates fertilization success remains unknown, and the mechanisms whereby intrinsic protein(s) in the ZP control the ZP meshwork of unfertilized eggs are still unclear. Recently, we found that dicalcin, a novel ZP protein-associated protein present in the intact VE of unfertilized eggs, suppresses fertilization to some degree by regulating the physiological properties of the VE through its association with gp41, a frog ortholog of ZP3^16^. Dicalcin belongs to the S100 protein family that includes twenty small (10–14 kDa) Ca^2+^-binding proteins, some of which are secreted from cells to exert their functions (*e.g*., S100B, S100A8/A9, and S100A12)^17^. Since S100 proteins lack an N-terminal leader sequence, they are probably secreted via as-yet-unknown mechanism different from the classic ER to Golgi pathway^18^. Dicalcin is present in the cytosol of the oocyte cortex besides the VE^16^, and possibly secreted from the oocytes into the frog egg-coating extracellular space. Note that leaderless secretion has been reported for other proteins such as interleukin 1β and fibroblast growth factor-2^19^. Our previous study demonstrated that the fertilization rate depends upon the amount of dicalcin in the VE: indeed, the fertilization rate increases as the amount of dicalcin decreases, and vice versa. Notably, this suppressive action occurs in unfertilized eggs at fertilization, which precedes the polyspermy block that occurs after fertilization. By harnessing this physiological function of dicalcin, we may be able to clamp the VE in a fertilization competent or incompetent status albeit still representing an extremely conditioned state. For example, when unfertilized eggs are treated with the peptide that corresponds to the gp41-binding region of dicalcin, the action of dicalcin may be enhanced, thereby rendering the VE status fertilization incompetent. In contrast, when unfertilized eggs are treated with another peptide that corresponds to the dicalcin-binding region of gp41, the action of dicalcin may be masked, allowing the VE status to be fertilization competent (see Supplementary Fig. S1). Thus, the objectives of this study were to determine the dicalcin-gp41 binding mechanism and to elucidate the fine structural basis for fertilization competence of the egg-coating envelope by utilizing synthetic peptides derived from two proteins. These aims were accomplished by biochemical analyses using a series of mutant proteins and histochemical examination at the electron microscopic level.

To roughly map the binding region of dicalcin to gp41, we generated a set of deletion mutants of dicalcin (Fig. 1a,b) and examined their binding by blot overlay (also known as Far Western) analysis (Fig. 1c). The intensities of biotinylated recombinants that bound to gp41 were converted to the molar amount of bound recombinants by referring to the biotin-coupling efficiency of each recombinant (Supplementary Fig. S2a). As shown in Fig. 1c, biotinylated dicalcin mutants that were truncated at the N-terminal side of dicalcin (His-Δ160C, Δ71N) lost their binding activity to gp41, whereas biotinylated mutants truncated at the C-terminal side (Δ43C, Δ101C) retained their binding activities, suggesting that the binding region was located within its N-terminal half of dicalcin, probably within region 2 (Fig. 1d,e). Unexpectedly, C-terminal-truncation mutants (Δ43C, Δ101C) exhibited greater binding activity than that of the wild-type protein (Fig. 1c), suggesting that dicalcin binding to gp41 was regulated by an intramolecular inhibitory mechanism (Fig. 1e, see details in Supplementary Fig. S3). To specify the binding region, we synthesized biotinylated peptides that flanked the N-terminal half of dicalcin (dcp1-dcp7 in Fig. 1f) and examined their binding to gp41. Among the examined peptides, two peptides (dcp4 and dcp7) had substantial binding activities and one peptide (dcp3) had a moderate binding activity (Fig. 1f). To further dissect these regions, we divided the above two peptides (dcp4 and dcp7) into shorter peptides (Fig. 1g) and measured their binding activities. Among them, dcp11 (S-F-S-C-N-Q-K-N-K) and dcp15 (A-A-L-C-K-L) exhibited the maximal binding activities to gp41, consistent with our early estimation that the primary binding site(s) may be localized within region 2 (Fig. 1d). Modeling of the three-dimensional structure of dicalcin^20^ revealed that these two regions were mapped to adjacent positions on the surface of the molecule (Supplementary Fig. S4), suggesting that these two regions acted coordinately.

Although these data indicated that the two amino acid regions are *in vitro* high-affinity binding sites for gp41, the roles of these regions in the suppression of fertilization by dicalcin were still uncertain. Therefore, we examined the effects of these peptides (dcp11 and dcp15) on *in vitro* fertilization. Pretreatment of the VE with the above two peptides (at 4 μM) markedly inhibited the efficiency of fertilization (dcp11, 25% of control, n = 7, p = 0.009; dcp15, 32% of control, n = 7, p = 0.01; Student’s *t*-test; Fig. 1h) in a concentration-dependent manner with submicromolar K_d_ values (33 nM for dcp11, 46 nM for dcp15, n = 15, Fig. 1i). Combined pretreatment with both peptides (at a 1:1 molar ratio) inhibited the fertilization rate (20% of control, n = 7, p = 0.006, Fig. 1h), exhibiting a concentration-dependent effect similar to that observed in each single pretreatment (Supplementary Fig. S4). These data indicated that the suppressive effects of both peptides were additive, but not synergistic and, therefore, binding sites for each peptide are likely to function independently and would not be expected to act mutually or allosterically. Thus, our results showed that these two regions were primary sites essential for the action of dicalcin.

The frog ZP3 protein, gp41, is secreted from oocytes, hydrolyzed by oviductin, a protease in the frog oviduct, and converted to mature gp41, ultimately resulting in the formation of ZP filaments^21^,^22^. In *X. laevis*, most sperm binding activity (~70%) is ascribed to gp41 and the rest is ascribed to gp64, a frog ZP2 ortholog^23^. The sequence of gp41 contains a single ZP domain (residues 10–294)^24^ that occupies ~90% of the full length of mature gp41. Recent X-ray crystallographic evidence of ZP proteins demonstrates that this ZP domain is divided into two domains (N-terminal ZP-N and C-terminal ZP-C), each of which is considered to function in the dimerization of ZP proteins^6^. In particular, ZP-C mediates the specific interactions between different ZP proteins during polymerization as well as species-restricted sperm-binding^6^. To examine whether dicalcin binds to the ZP domain of gp41 or another region of gp41, we generated full-length gp41, ZP-N (residues 10-110) and ZP-C (residues 136–294) domains (Fig. 2a) and investigated their binding to dicalcin and two dicalcin-derived peptides. The wild-type dicalcin and the two dicalcin-derived peptides (dcp11 and dcp15) bound to both full-length and the ZP-C domain of gp41 (Fig. 2b), indicating that the potential binding site(s) resides in the ZP-C domain. To map the dicalcin-binding sites within ZP-C, we generated a set of ZP-C truncated mutants and examined their binding to dicalcin-derived peptides (Fig. 2c). Both biotinylated dicalcin-derived peptides showed the binding activity to His-Δ76C (truncation of the C-terminal side of ZP-C) (Fig. 2d), the degree of which was similar to that of wild-type ZP-C (Fig. 2e). Neither peptide showed binding to His-Δ89C or His-Δ63N (truncation of the N-terminal side of ZP-C), indicating that the binding site for both peptides was located within amino acid residues 186–218 of ZP-C (Fig. 2c).

To further characterize this region (residues 186–218), we divided this region into two regions and examined the binding of the synthesized peptides to dicalcin. Of the two peptides, gpp2 (R-Y-E-I-I-N-Q-N-G-C-L-V-D-G-K-L-D-D-S-S-S-A-F) bound to dicalcin (Fig. 2f), and significantly antagonized the binding of dicalcin-derived peptides to gp41 (58% reduction for dpc11, 55% for dpc15; Fig. 2g). Surprisingly, modeling of the three-dimensional structure of gp41 revealed that this region was mapped to the surface of the molecule at a position adjacent to the dimerization interface, and extended into a part of the ZP-C subdomain that is considered to be important for the multiple roles of gp41 (residues 250–256 and 312–343, Fig. 2i). We next examined the effect of gpp2 on *in vitro* fertilization to assess a role of this region in mediating the action of dicalcin, and found that preincubation of the VE with gpp2 increased the efficiency of fertilization in a concentration-dependent manner (Fig. 2h). In particular, preincubation of gpp2 at a concentration of 4 μM enhanced fertilization up to 132% of the control (n = 30–40, mean ± s.e.m., *p = 0.031. **p = 0.015, Student’s *t*-test). Note that the actual fertilization rate of dejellied eggs was reduced to ~40% in our assay, possibly due to the absence of jelly layer. This actual fertilization rate under control was set to 100% in each trial, and data were normalized. Taken together, these results showed that this region was an essential binding site of gp41 and was required for mediating the action of dicalcin.

Carbohydrate-dependent recognition has been repeatedly implicated to play an important role for establishment of an appropriate sperm-egg interaction^25^. Indeed, alterations in the staining pattern of a lectin have been observed in human ZP of fertilization-failed oocytes^15^. Our previous study has also revealed that pretreatment with dicalcin increases *in vivo* reactivity of the VE to the Gal/GalNAc-sensitive lectin, *Ricinus communis* agglutinin I (RCAI), suggesting that dicalcin regulates the distribution pattern of oligosaccharides within the VE through its binding to gp41^16^. To verify whether dicalcin- and gp41-derived peptides also alter the distribution pattern of oligosaccharides, we examined the lectin reactivity of the VE following peptide pretreatment. Consistent with our previous study, RCAI reacted only with gp41 in our lectin blot analysis (Fig. 3a), and its reactivity increased following pretreatment with dicalcin when RCAI-staining signals were quantified across the VE (Fig. 3b). Pretreatment with dcp11 augmented RCAI reactivity to the same extent as with dicalcin pretreatment (Fig. 3c), indicating that dicalcin and dcp11 caused similar changes in the distribution pattern of the RCAI ligand.

Since pretreatment with gpp2 did not alter RCAI reactivity (Fig. 3d), we sought another lectin that may detect potential alterations in sugar-distribution pattern induced by gpp2 pretreatment. The GlcNAc/sialic acid-sensitive lectin, wheat germ agglutinin (WGA) was found to react with gp41, gp120, and gp69 in the lectin blot analysis (Fig. 3e). As observed in RCAI staining, pretreatment with dicalcin and dcp11 caused similar alterations in WGA staining (Fig. 3f,g). In addition, WGA staining was altered in response to gpp2 pretreatment: pretreatment with gpp2 abolished WGA reactivity in the outermost zone and shifted the midsectional reactive zone toward the egg-plasma membrane (Fig. 3h). Note that this complex WGA staining pattern is possibly due to its broad reactivity: WGA reacted with the above three glycoproteins, but not with gp41 alone as observed in the RCAI staining (Fig. 3a). These results demonstrated that dcp11 and dicalcin elicited a similar three-dimensional alteration in the oligosaccharide-distribution pattern and three-dimensional meshwork of the VE as well, and that the gp41-derived peptide induced a distinct alteration in the three-dimensional structure. Furthermore, our data confirmed that it was possible to precisely control the fertilization competence of the VE of unfertilized eggs by utilizing these two peptides.

To investigate the fine structural basis underlying the three-dimensional alterations described above, we next used transmission electron microscopy (TEM). TEM analysis revealed that ZP filaments pretreated with dcp11 were arranged parallel to the egg plasma membrane, giving the appearance of a “pin-stripe” pattern (Fig. 4b, dcp11). On the other hand, ZP filaments treated with gpp2 were randomized and many filaments were arranged oblique to the egg plasma membrane, forming a zigzag or occasionally “herring-bone” pattern (Fig.4b, gpp2). These results suggested that treatment with dcp11 and gpp2 caused two distinct nanoscale meshworks that determined whether the VE was fertilization competent (caused by gpp2) or incompetent (caused by dcp11). These structural differences of the VE likely underlie the suppressive action of dicalcin on sperm-binding and sperm-penetration processes in frogs^16^ and may also be responsible for the variations in fertility observed in many species.

We further examined whether the above two states could switch by repeated treatment of the VE: dcp11 treatment following gpp2 pretreatment (gpp2 → dcp11) or gpp2 treatment following dcp11 pretreatment (dcp11 → gpp2). Treatment with gpp2 → dcp11 augmented RCAI reactivity to a similar extent as with dcp11 treatment alone, whereas treatment with dc11 → gpp2 did not alter RCAI reactivity, similar to gpp2 treatment alone (Fig. 5a). As for the WGA ligand, the staining patterns of gpp2 → dc11 and dc11 → gpp2 were similar to those of dcp11 or gpp2 treatment alone, respectively (Supplementary Fig. S5). These results demonstrated that the peptide-induced fertilization competent status could be converted, and therefore fertilization competent state has a structural plasticity determined by the dicalcin-gp41 interaction. A schematic representation describing the possible three-dimensional meshwork model of ZP filaments regarding fertilization competence is shown in Fig. 5b.

Characterization of the fertilization competence of the egg-coating envelope is of great importance to our understanding of the molecular mechanisms underlying the fertilization success. Our present study identified interactive regions between dicalcin and gp41 and demonstrated that dicalcin- and gp41-derived peptides were capable of controlling the fertilization success. Under peptide-controlled conditions, the nanoscale structure of the VE was markedly different between eggs exhibiting fertilization-competent and -incompetent statuses, providing strong evidence for a structural basis of the fertilization competence in the VE. The data presented here are also the first to show reversible transition of the fertilization competence by extrinsic treatment, and interestingly mouse dicalcin has also been reported to inhibit *in vitro* fertilization^26^. Therefore our study may promote the development of efficacious drugs (*e.g*. for *in utero* administration) for contraceptive strategy and the treatment of infertility in mammals including domestic animals and even humans.

## Additional Information

**How to cite this article**: Miwa, N. *et al.* Fertilization competence of the egg-coating envelope is regulated by direct interaction of dicalcin and gp41, the *Xenopus laevis* ZP3. *Sci. Rep.* **5**, 12672; doi: 10.1038/srep12672 (2015).

## Supplementary Material

Supplementary Information

## Figures and Tables

**Figure 1:**
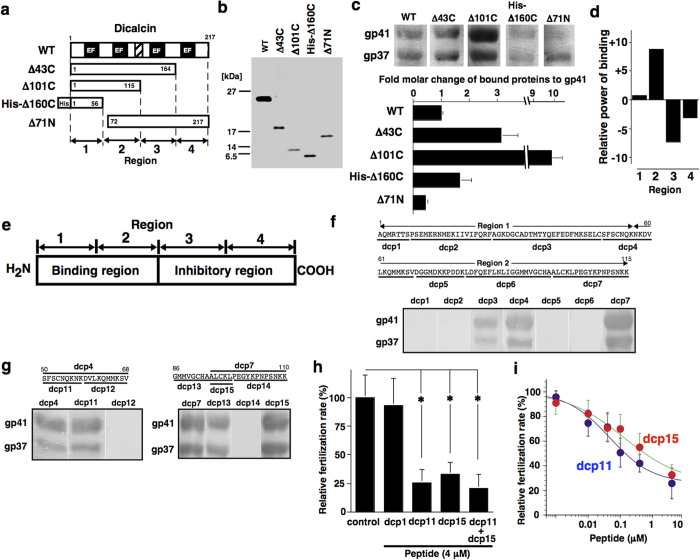
Identification of the amino acid regions mediating the suppressive action of dicalcin on fertilization. **(a)** A schematic representation of deleted mutants of dicalcin. The primary structure of dicalcin contains four calcium-binding domains (EF-hand) and linker region (hatched) that links the N-terminal and C-terminal halves. The *numbers* in the boxes refer to the amino acid positions. One mutant (Δ160C) was histidine-tagged at the N-terminus. On the basis of deleted regions, the sequence of dicalcin was divided into four regions (regions 1, 2, 3 and 4). **(b)** Purification of wild type and mutants of dicalcin. Purified recombinants were electrophoresed and silver-stained. **(c)** Binding of wild type and mutants to gp41 and gp37. Blots of VE proteins were probed by biotinylated proteins. Intensities for gp41 were converted to the molar amounts of bound proteins. The molar amount of the wild-type was set to 100% and the data were normalized (n = 10, mean ± s.e.m). **(d)** Binding activities of four regions of dicalcin. Binding activities of each region were estimated by referring to normalized binding activities in **(c)**. **(e)** A schematic diagram of the role of each region for gp41-binding. N-terminal region of dicalcin is required for gp41-binding, whereas C-terminal region suppresses the binding (see Supplementary Fig. S3). **(f)** Binding of the synthetic peptides on gp41 and gp37. The sequence of N-terminal region (regions 1 and 2) was divided into seven regions. Biotin-coupled peptides corresponding to each region were probed on VE proteins. **(g)** Binding of shorter peptides on gp41 and gp37. (Left) The amino acid region of dcp4 was divided into two regions (dcp11 and dcp12). (Right) The amino acid region containing dcp7 was divided into three regions (dcp13, dcp14, and dcp15). **(h)** Effect of pretreatment with dicalcin-derived peptides on the fertilization rate. Ovulated eggs were pretreated with BSA (control), peptides (dcp1, dcp11, dcp15 and 1:1 mixture of dcp11 and dcp15) followed by insemination. The fertilization rate was scored as percentage of fertilized eggs/all eggs and normalized (n = 7; *p < 0.02, Student’s *t*-test). **(i)** Concentration dependence of inhibitory effect of dcp11 and dcp15. The graph shows mean data (n = 10, mean ± s.e.m).

**Figure 2:**
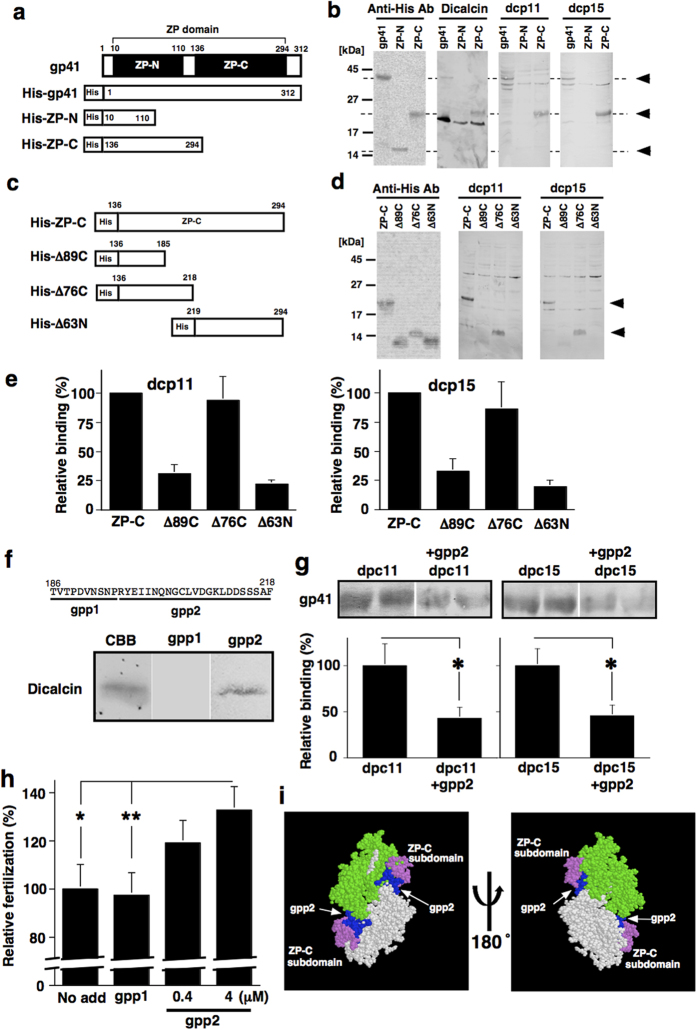
Identification of amino acid region of gp41 essential in mediating the action of dicalcin. **(a)** A schematic representation of the wild-type, ZP-N and ZP–C domains of gp41. The *numbers* in the boxes refer to the amino acid positions. All recombinants were histidine-tagged at the N-terminus. **(b)** Binding activities of each mutant to dicalcin-derived peptides. Each of the wild type and mutants was expressed in *E. coli*, and the blots of their lysates were probed either by anti-histidine antibody (Anti-His Ab), biotinylated dicalcin (Dicalcin), dcp11, or dcp15. Arrowheads indicated positions of recombinants for the wild type, ZP-N, and ZP-C. **(c)** A schematic representation of ZP-C and deleted mutants. **(d)** Binding of dicalcin-derived peptides to each mutant. Each of the wild type and mutants was expressed in *E. coli*, and the blots of their lysates were probed by anti-histidine antibody (Anti-His Ab), dcp11, or dcp15. **(e)** The binding activities of dicalcin-derived peptides to ZP-C and mutants. Intensities of dcp11- (Left) and dcp15- (Right) binding to mutants were normalized by those to ZP-C (n = 10, mean ± s.e.m). **(f)** Binding of gp41-derived peptide on dicalcin. The amino acid sequence (residues 486–518) was divided into two regions (gpp1 and gpp2), and their binding to dicalcin was examined. CBB, CBB-stained dicalcin; gpp1, treated with biotinylated gpp1; gpp2, treated with biotinylated gpp2. **(g)** Inhibition of the binding of dicalcin-derived peptides to gp41 by gpp2. Blots of VE proteins were probed with biotinylated dicalcin-derived peptides (dcp11 and dcp15, 1 μM) either in the presence (1 μM) or absence of gpp2. Co-incubation with gpp2 reduced the binding (n = 10; *p < 0.03, Student’s *t*-test). **(h)** Effect of pretreatment with gpp2 on fertilization. Normalized fertilization rate increased up to 132% of control (n = 30–40, mean ± s.e.m., *p = 0.031. **p = 0.015, Student’s *t*-test). **(i)** Mapping of the amino acid region of gp41 in mediating the action of dicalcin. Each monomer of gp41 was shown in green and white. The region for gpp2 was shown in blue (residues 242–264 in each monomer of chicken ZP3). ZP-C subdomain was shown in violet (residues 250–256, and 312–343).

**Figure 3:**
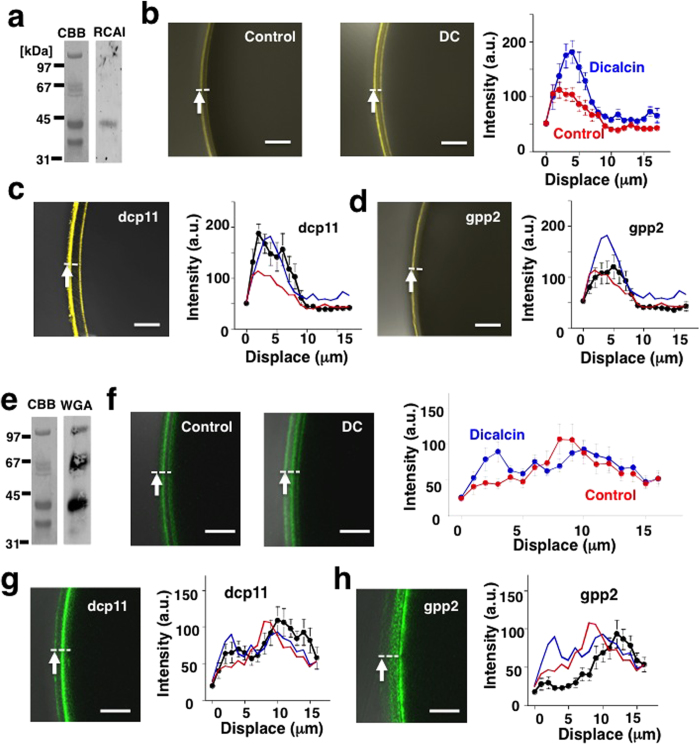
Dicalcin- and gp41-derived peptides induce alterations in *in vivo* lectin reactivities of the VE. (**a**) Blot of VE proteins treated with RCAI. CBB, CBB-stained VE; RCAI, Rhodamine-labeled RCAI blot. (**b**) Representative confocal images of a *Xenopus* egg treated with RCAI and averaged intensities across the VE. (Left) RCAI stained the outermost region of the VE following treatment with BSA (Control) or dicalcin (DC). Scale bar: 50 μm. (Right) Intensities of RCAI staining across the VE (dashed line in Control in the left, n = 15). The position where RCAI-signal starts to rise is designated as 0 μm in the x axis (arrow in the left). **(c)** RCAI staining of the VE pretreated with dcp11. (Left) RCAI reactivity of the VE pretreated with dcp11. (Right) Intensities across the VE (black) (n = 15). RCAI reactivities pretreated with dicalcin (blue) and BSA (red) were also shown. (**d**) RCAI staining of the VE pretreated with gpp2. (Left) RCAI reactivity of the VE pretreated with gpp2. (Right) Intensities of the VE across the VE (black) (n = 15). (**e**) Blot of VE proteins treated with WGA. WGA recognized gp41 as well as gp69/64 and gp120. CBB, CBB-stained VE; WGA, FITC-labeled WGA blot. (**f**) Representative confocal images of a *Xenopus* egg treated with WGA and averaged intensities across the VE. (Left) WGA stained the outermost and midsection regions of the VE following treatment with BSA (Control) or dicalcin (DC). Scale bar: 50 μm. (Right) Intensities of WGA staining across the VE (n = 15). The position where WGA-signal starts to rise is designated as 0 μm in the x axis (arrow in the left). (**g**) WGA staining of the VE pretreated with dcp11. (Left) WGA reactivity of the VE pretreated with dcp11. (Right) Intensities of the VE across the VE (black) (n = 15). WGA reactivities of the VE pretreated with dicalcin (blue) and BSA (red) were also shown. **(h)** WGA staining of the VE pretreated with gpp2. (Left) WGA reactivity of the VE pretreated with gpp2. (Right) Intensities of the VE across the VE (black) (n = 15).

**Figure 4:**
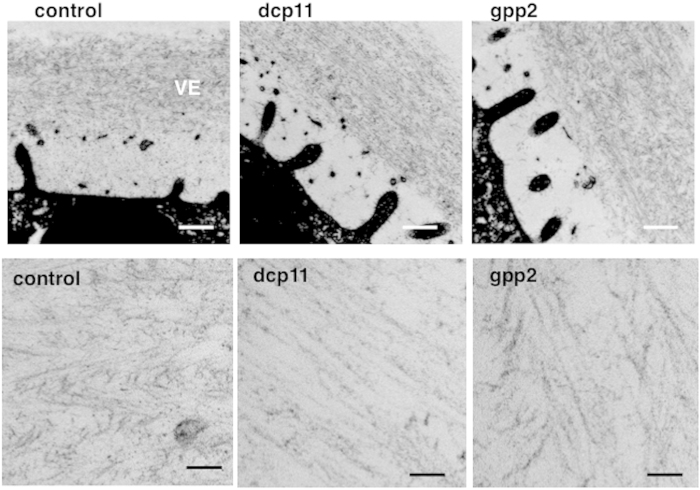
Dicalcin-and gp41-derived peptides induce distinct nanoscale ZP meshworks. TEM analysis of the VE treated with dcp11 and gpp2. (Upper) Low magnified images of the VE treated with peptides (dcp1 as control, dcp11 and gpp2; 4 μM; n = 3). Note that intensities were enhanced to obtain fine details of ZP filaments. Scale bar: 500 nm. (Lower) Higher magnified images. Scale bar: 30 nm.

**Figure 5:**
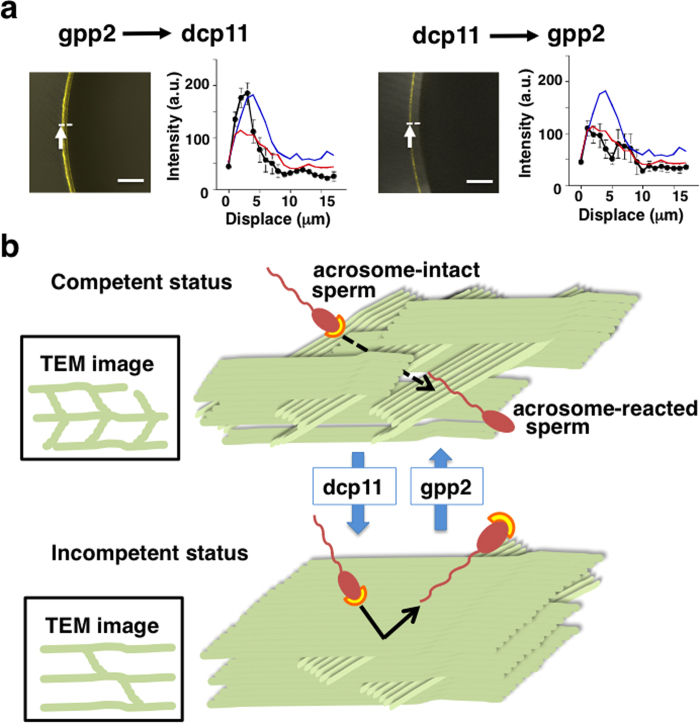
Reversible transition of the ZP meshwork status by substitution treatment with dcp11 and gpp2. (**a**) RCAI staining of the VE substitutionally treated with dcp11 and gpp2. gpp2 → dcp11; eggs were pretreated first with gpp2 (4 μM), followed by rinse and treatment with dcp11 (8 μM). dcp11 → gpp2; eggs were pretreated first with dcp11 (4 μM), followed by rinse and treatment with gpp2 (8 μM). (Left) Representative confocal image of unfertilized egg. (Right) Intensities across the VE (black) (n = 15). RCAI reactivity of the VE pretreated with dcp11 (blue) and gpp2 (red) were also shown. (**b**) A schematic model of the transition of ZP meshwork between fertilization competent and incompetent statuses. Our TEM studies revealed that ZP filaments pretreated with dcp11 were arranged parallel to the egg plasma membrane, exhibiting a “pin-stripe” pattern, while ZP filaments pretreated with gpp2 were arranged oblique to the egg plasma membrane, occasionally forming a “herring-bone” pattern. These results implies that the ZP filaments pretreated with dcp11 was a well-organized sheet-like structure, while the ZP filaments pretreated with gpp2 was randomly disoriented organization. On the basis of these two-dimensional images, we hypothesized three-dimensional meshwork model of ZP filaments. Treatment with gpp2 induces a randomized disoriented ZP meshwork that allows sperm to fit into the three-dimensional structure (*i.e.* capture of sperm), and enables acrosome reaction, while treatment with dcp11 alters a better-organized meshwork, forming parallel sheet of ZP filaments where sperm may not fit to the structure and therefore sperm move away from the VE, resulting in fertilization failure.
