# Corrigendum: Fertilization competence of the egg-coating envelope is regulated by direct interaction of dicalcin and gp41, the *Xenopus laevis* ZP3

**DOI:** 10.1038/srep15303

**Published:** 2015-10-21

**Authors:** Naofumi Miwa, Motoyuki Ogawa, Mayu Hanaue, Ken Takamatsu

Scientific Reports 5: Article number 12672; 10.1038/srep12672 published online: 07052015; updated: 10212015.

The original version of this Article contained typographical errors.

In the Abstract,

“The interactive regions between dicalcin and gp41 comprised six and nine amino acid residues within dicalcin and twenty-three within gp41.”

now reads:

“The interactive regions between dicalcin and gp41 comprised five and nine amino acid residues within dicalcin and twenty-three within gp41.”

In the Introduction,

“Among them, dcp11 (S-F-S-C-N-Q-K-N-K) and dcp15 (A-A-L-C-K-L) exhibited the maximal binding activities to gp41, consistent with our early estimation that the primary binding site(s) may be localized within region 2 (Fig. 1d).”

now reads:

“Among them, dcp11 (S-F-S-C-N-Q-K-N-K) and dcp15 (A-L-C-K-L) exhibited the maximal binding activities to gp41, consistent with our early estimation that the primary binding site(s) may be localized within region 2 (Fig. 1d).”

These errors have now been corrected in both the PDF and HTML versions of the Article.

