# Supplementary material for: Fertilization competence of the egg-coating envelope is regulated by direct interaction of dicalcin and gp41, the Xenopus laevis ZP3
Source: Sci Rep. 2015 Aug 5;5:12672. doi: 10.1038/srep12672 (PMC4525147; doi:10.1038/srep12672)
Supplement: Supplementary Information [file srep12672-s1.pdf]

Supplementary Information

Fertilization competence of the egg-coating envelope is regulated by direct interaction of dicalcin and gp41, the *Xenopus laevis* ZP3.

Naofumi Miwa[1], Motoyuki Ogawa[2], Mayu Hanaue[1], Ken Takamatsu[1]

[1]*Department of Physiology, School of Medicine, Toho University, Ohmori-nishi 5-21-16, Ohta-ku, Tokyo 143-8540, Japan.* [2]*Department of Anatomy, School of Medicine, Kitasato University, Kitasato 1-15-1, Sagamihara, Kanagawa 228-8555, Japan.*

Methods

Expression of the wild type and mutants of dicalcin in *E.coli*. Three of deletion mutants (Δ43C, Δ101C, Δ160C) were truncations of 43, 101 and 160 amino acids from the C-terminus of dicalcin, whereas the other mutant (Δ71N) was truncation of 71 amino acids from the N terminus. The full-length coding region of dicalcin and DNA fragments corresponding to residues 1-164 for Δ43C, 1-115 for Δ101C, 1-56 for Δ160C, 72-217 for Δ71N were PCR-amplified, ligated with pET-3a (Novagen, EMD Biosciences, San Diego, CA) and introduced into *E.coli* BL21 pLysS (Novagen). Primer informations are seen below. Recombinant wild-type dicalcin and deletion mutants were expressed and purified according to procedures as described previously[1]. Since Δ160C mutant was not applicable to the above purification procedure due to low affinity with a phenyl-Sepharose resin (Amersham), the corresponding DNA fragment was, in turn,

ligated with pET-16b (Novagen, EMD Biosciences, San Diego, CA) to add histidine-tag into the N-terminus of the mutant, and purified using metal-affinity column chromatography according to procedures shown elsewhere.

VE protein preparation. VE proteins of *Xenopus* unfertilized eggs were prepared by sieving method described elsewhere[2,3]. Briefly, envelopes were collected by passing dejellied egg-lysate through a nylon filter and the filter was washed extensively with distilled water. Isolated envelopes were stored overnight in 2M NaCl, 2 mM $CaCl_2$, 10 mM Tris-HCl (pH 7.4) to selectively solubilize contaminating yolk platelets[4], and heated at 70 °C before use.

Blot overlay analysis and estimation of binding activities of each region. Blot overlay analyses using biotinylated dicalcin, its mutants and synthetic peptides were performed according to our previous method[5]. Briefly, VE proteins were electrophoresed and blotted onto a PVDF membrane. After renaturing and blocking, blots were incubated with biotinylated recombinants and synthetic peptides (final concentration, 1 μM) overnight at 4 °C. After wash, biotinylated recombinants and peptides bound to a target protein was coupled to HRP using a Vectastain ABC kit (Vector Laboratories, Burlingame, CA) and visualized. Intensities of blots were converted to the molar amount of bound proteins on the basis of the coupling efficiency of biotinylation. The molar amount of the wild-type was set to 100% and the data were normalized. Binding activities of each region of dicalcin were estimated referring to the

normalized fold change of the molar amount. For example, the activity of region 1 of dicalcin was the subtraction of the relative molar amount of Δ71N from that of the wild type, and the activity of region 2 was the subtraction of the relative molar amount of His-Δ160C from that of Δ101C.

Expression of the wild type and mutants of gp41 in *E.coli*. The full-length coding region of mature gp41 and DNA fragments corresponding to the ZP-N (residues 10-110) and ZP-C (residues 136-294) domains were PCR-amplified. Primer informations are seen below, together with those for dicalcin-mutants. The sequences of fragments were verified by DNA sequencing, subsequently ligated with pET-16b, and introduced into *E.coli* BL21 pLysS. For deletion mutants of the ZP-C domain, two mutants (Δ89C, Δ76C) were truncations of 109, 72 amino acids from the C-terminus of ZP-C domain of gp41, while the other mutant (Δ63N) was truncation of 73 amino acids from the N terminus. DNA fragments corresponding to residues 136-185 for His-Δ89C, 136-218 for His-Δ76C, 219-294 for His-Δ63N were PCR-amplified, verified by DNA sequencing, inserted into XhoI site of pET-16b and introduced into *E.coli* BL21 pLysS. Schematic diagrams for the above mutants were shown in Fig. 2a and d.

Modeling of three-dimensional structure of dicalcin and gp41.

The graphics program RASMOL was used for modeling of three-dimensional structure of dicalcin already reported in our previous study[6] and the structure of *Xenopus* gp41 was adapted from chicken ZP3 (3NK4).

Preparation of gametes. *Xenopus* gametes were prepared as previously described[7]. Briefly, freshly squeezed eggs were immediately washed three times with MMR solution (100mM NaCl, 2mM KCl, 2 mM $CaCl_2$, 0.1mM EDTA, 1mM $MgSO_4$, 5 mM Hepes; pH 7.4). Jelly was removed by exposure (5min) of the eggs to 2% cystein. The resultant jelly-free eggs were gently washed with several times by 0.3xMMR. Jelly extract was prepared as described elsewhere[8]. Jelly-coated eggs were subjected to rocking with 10mL of 0.3xMMR for 60 min and the medium was mixed with Ficoll (final concentration, 10%; Sigma, St. Louis, MO), providing "jelly-extract". Sperm were prepared from freshly excised testis in 1mL of 0.3xMMR. The intact sperm were diluted in the jelly extract.

***In vitro* fertilization assay.** *In vitro* fertilization assay was also performed as previously described[7]. Briefly, dejellied eggs in 0.3xMMR of ~500 μL were pre-incubated with the following material for 15min; dicalcin and peptides at indicated concentrations, BSA (4 μM). After washing, dejellied eggs were inseminated with sperm (final concentration:~$5x10^6$/mL) treated with jelly-extract. The successful fertilization was scored by counting eggs that underwent first cleavage until when some of fertilized eggs proceeded to the 4-cell-stage. All counts were done blind to conditions.

Lectin blot analyses and lectin cytochemistry. VE proteins were electrophoresed and blotted onto a PVDF membrane. After blocking with synthetic polymers (PVDF

Blocking Reagent, TOYOBO, Osaka, Japan), blots were probed with 50 μg/mL lectin conjugates (Rhodamine-RCAI, Fluorescein-WGA; Vector Labs, Burlingame CA). After wash, bound lectin was visualized by the fluorescence scanner (Typhoon 9400, GE healthcare). For lectin cytochemistry, unfertilized eggs were pretreated with dicalcin, peptides or BSA (4 μM each) in 0.3xMMR at room temperature for 15 min. After wash, eggs were treated either with Rhodamine-labeled RCAI or Fluorescein-WGA (50 μg/mL) for 30min and analyzed using a confocal microscope. All examinations were done blind to conditions.

Electron microscopic analyses. Dejellied *Xenopus* were pre-incubated with either of peptides (dcp1, dcp11 and gpp2) at a concentration of 4 μM for 15 min, and fixed in 2% paraformaldehyde overnight at 4 °C. Glutaraldehyde-fixed egg was refixed with 2% OsO_4 and dehydrated with a graded series (30-100%) of ethanol. Gelatine capsules containing epoxy resin (Quetol 812, Nisshin-EM, Tokyo, Japan) were inverted onto the specimens. After the resin was cured at 60 for 48hr, the capsules were detached. Ultrathin sections (~80 nm thickness) were stained with uranyl acetate and lead acetate, and observed with a Hitachi H-7600 (Hitachi, Tokyo, Japan) at 100kV.

Primers.

For generation of deleted-mutants, we amplified cDNA of dicalcin and gp41 by PCR using primes stated as below. The oligonucleotides for forward and reverse primers were synthesized with NdeI and BamHI sites on their 5’ ends, respectively. Their

sequences were as follows:

Wild-type dicalcin, 5’-GCCCAGATGCGCA-3’ and 5’-TCAGGGTTTCTTGGGGT-3’; Δ43C, 5’-GCCCAGATGCGCA-3’ and 5’-TCACTTCTGGCCCCTCGGTG-3’; Δ101C 5’-GCCCAGATGCGCA-3’ and 5’-TCACTTCTTGTTAGAGGGGT-3’; His-Δ160C 5’-GCCCAGATGCGC-3’ and 5’-TCACTTCTGGTTACAGGAGA-3’; Δ71N 5’-GACAAGAAACCA-3’ 5’-TCAGGGTTTCTTGGGGT-3’. A schematic diagram of the mutants is shown in Fig. 1a. PCR-amplified fragments were verified by DNA sequencing and fragments of Δ43C, Δ101C and Δ71N were inserted into NdeI/BamHI sites of pET-3a, whereas the fragment of Δ160C was into NdeI/BamHI sites of pET-16b to obtain His-Δ160C. For generation of gp41-mutants, the oligonucleotides for forward and reverse primers were synthesized with XhoI site on their 5’ ends. Their sequences were as follows: His-gp41, 5’-CAACTCCCCGTCTCCCCCC-3’ and 5’-TCACCTAGAGCCTGAAAAATAT-3’; His-ZP-N, 5’-GTCAGTGTGTGCGATGTGAT-3’ and 5’-TCACCTGGGATAAAAGCA -3’; His-ZP-C,5’-CTAGCATTCTCATTACGCC-3’and5’-TCAACAGTTTCCAGTATCA-3’; His-Δ89C, 5’-CTAGCATTCTCATTACGCC-3’ and 5’-TCAGGCCACACAGCTGTC-3’; His-Δ76C, 5’-CTAGCATTCTCATTACGCC-3’ and 5’-TCAGAAGGCAGAAGAAGAGT-3’; His-Δ63N, 5’-CGATCTCCAAGGCCCCA-3’, 5’-TCAACAGTTTCCAGTATCA-3’. A schematic diagram of the mutants is shown in Fig. 2a and c. PCR-amplified fragments were verified by DNA sequencing and inserted into XhoI site of pET-16b.

Peptides. Peptides that correspond to dicalcin and gp41 sequences were synthesized, purified and biotinylated by BEX (Tokyo, Japan). The purity of each peptide was >95%. Peptides were diluted in 0.3xMMR to indicated concentrations for use. At preparation of peptides, we determined the length of a peptide to keep α-helices of each peptide as intact as possible.

Animal care. All animal experiments were approved and in accordance with the animal care committee's at Toho University.

References

1. Miwa, N., Kobayashi, M., Takamatsu, K. & Kawamura, S. *Biochem. Biophys. Res. Commun.* **251**, 35-40 (1998)

2. Wolf, D.P., Nishihara, T., West, D.M. & Hedrick, J.L. *Biochemistry* **15**, 3671-8 (1976)

3. Richter, H.P. *Cell Biol. Int. Rep.* **4**, 985-995 (1980)

4. Lindsay LL., & Hedrick JL. *J. Exp. Zool.* **245**, 286-293 (1988)

5. Miwa, N., Uebi, T. & Kawamura, S. *J. Biol. Chem.* **275**, 27245-9 (2000)

6. Tanaka T. *et al. Protein Eng.* **12** 395-405 (1999)

7. Miwa N. *et al. J.Biol.Chem.* **285**, 15627-26 (2010)

8. Heasman, J. Holwill, S. & Wylie, C.C. *Methods Cell Biol.* **36**, 213-30 (1991)

Supplementary Figure Legends

Supplementary Figure S1 Assumed model of the control of fertilization success by dicalcin- and gp41-derived peptides.

(a) Model of the interaction between dicalcin and gp41. The direct binding of dicalcin and gp41 involves the interactive amino acid regions of dicalcin and gp41. Our previous results have demonstrated that dicalcin-binding to gp41 induces an alteration in the configuration of oligosaccharide on the gp41 surface, which is related with an inhibitory action of dicalcin on fertilization success[5]. Therefore, it is assumed that dicalcin binds to gp41 via potential binding region to gp41, and leads to its conformational change.

(b) A case of unfertilized eggs pretreated with excess amounts of the peptide that corresponds to the gp41-binding region of dicalcin. When unfertilized eggs are pretreated with excess amounts of this peptide (red), the action of dicalcin is enhanced, and therefore the fertilization may result in failure.

(c) A case of unfertilized eggs pretreated with excess amounts of the peptide that corresponds to the dicalcin-binding region of gp41. When unfertilized eggs are pretreated with excess amounts of this peptide (blue), the action of dicalcin is masked, and therefore the fertilization may be facilitated.

Supplementary Figure S2 Region-specific binding properties of dicalcin to gp41 and gp37.

(a) Coupling efficiency of biotinylation to each recombinant. The degree of biotin-coupling was calibrated to the molar amounts of purified recombinants. The table shows the normalized coupling efficiency per mole of protein. **(b)** Intensities of

biotinylated wild type and mutants that bound to gp41. Blots of VE proteins were probed by biotinylated proteins and intensities of the blots were averaged (n=15, mean±s.e.m.) **(c)** Intensities of biotinylated wild type and mutants that bound to gp37. The amount of bound proteins was averaged (n=15, mean±s.e.m.). **(d)** Normalized molar amount of bound proteins to gp37. Intensities of blots were converted to the molar amount of bound proteins. The molar amount of the wild-type was set to 100% and the data were normalized. **(e)** Binding ability of four regions of dicalcin to gp37. Binding activities of each region was calculated by subtraction of normalized binding activities (see Methods). **(f)** A schematic diagram of the role of each region for the binding to gp37. The region 2 only acts as the binding region, whereas the other regions (regions 1, 3 and 4) inherently inhibit the binding to gp37 (see Supplementary Fig. S3).

Supplementary Figure S3 Intramolecular inhibitory regulation of dicalcin-binding to gp41.

The C-terminal region of dicalcin hampers the binding activity of the wild type by suppressing the N-terminal region as shown in Fig. 1c and d. We examined whether a part of C-terminal region of dicalcin directly could interfere with the binding of N-terminal region to gp41. **(a)** The sequences of synthetic peptides of dicalcin. Partial sequence (residues 1-164) of dicalcin encompassing regions 1, 2 and 3 is divided into ten subregions. Region 3 was found to have the maximal inhibitory activity, whereas regions 1 and 2 play a role for the binding of wild type of dicalcin (Fig. 1d and e). Among synthetic peptides that correspond to the N-terminal half, two peptides (dcp4

and dcp7) have the greatest binding activities to gp41 in our blot overlay analysis (Fig. 1c). **(b)** Non-influence of peptides (dcp8-10) on dcp4-binding to gp41. Blots of VE proteins were probed with biotinylated dcp4 (1 μM) either in the presence (1 μM) or absence of dicalcin-derived peptides (dcp8, dcp9 and dcp10). The graph shows mean data (n=10, mean±s.e.m.). **(c)** Non-influence of peptides (dcp8-10) on dcp7-binding to gp41. Blots of VE proteins were probed with biotinylated dcp7 (1 μM) either in the presence (1 μM) or absence of dicalcin-derived peptides (dcp8, dcp9 and dcp10). The graph shows mean data (n=10, mean±s.e.m.). These results indicated that inhibitory mechanism of the C-terminal region of dicalcin is not the direct antagonism with the binding of the N-terminal region, but unknown intramolecular mechanism where the interaction between the C-terminal region and the N-terminal region may affect the circumstances (*e.g.* electrostatic conditions) around the binding region in the N-terminal half.

Supplementary Figure S4 Three-dimensional modeling of gp41-binding regions in dicalcin.

(a) Concentration-dependent effect of pretreatment with combined dicalcin-derived peptides on the efficiency of fertilization *in vitro*. Ovulated eggs were pretreated with peptides (at a 1:1 mixture of dcp11 and dcp15) followed by incubation of sperm. Fertilization success was scored and normalized (n=7; *, p=0.007, Student's *t*-test). Concentration-dependence curves at pretreatments with dcp11(blue) and dcp15 (red) alone were also shown. **(b)** Mapping of the amino acid region of dicalcin for its binding

to gp41. Locations of dcp11 (residues 50-58, red) and dcp15 (residues 98-103, yellow) were mapped by spacefill (upper) and ribbon (lower) models of dicalcin[6]. Amino acid regions of dcp11 and 15 were mapped to an adjacent position, and were suggested to form a loop (dcp11) and short α-helix (dcp15) structures.

Supplementary Figure S5 Alterations in WGA-staining of the VE substitutionally pretreated with dcp11 and gpp2.

gpp2→dcp11; eggs were pretreated first with gpp2 (4 μM), followed rinse and treated with dcp11 (8 μM). dcp11→gpp2; eggs were pretreated first with dcp11 (4 μM), followed rinse and treated with gpp2 (8 μM). (Left) Representative confocal image of unfertilized egg. (Right) Intensities across the VE (black) (n=15). WGA reactivities of the VE pretreated with dcp11 (blue) and gpp2 (red) were also shown.

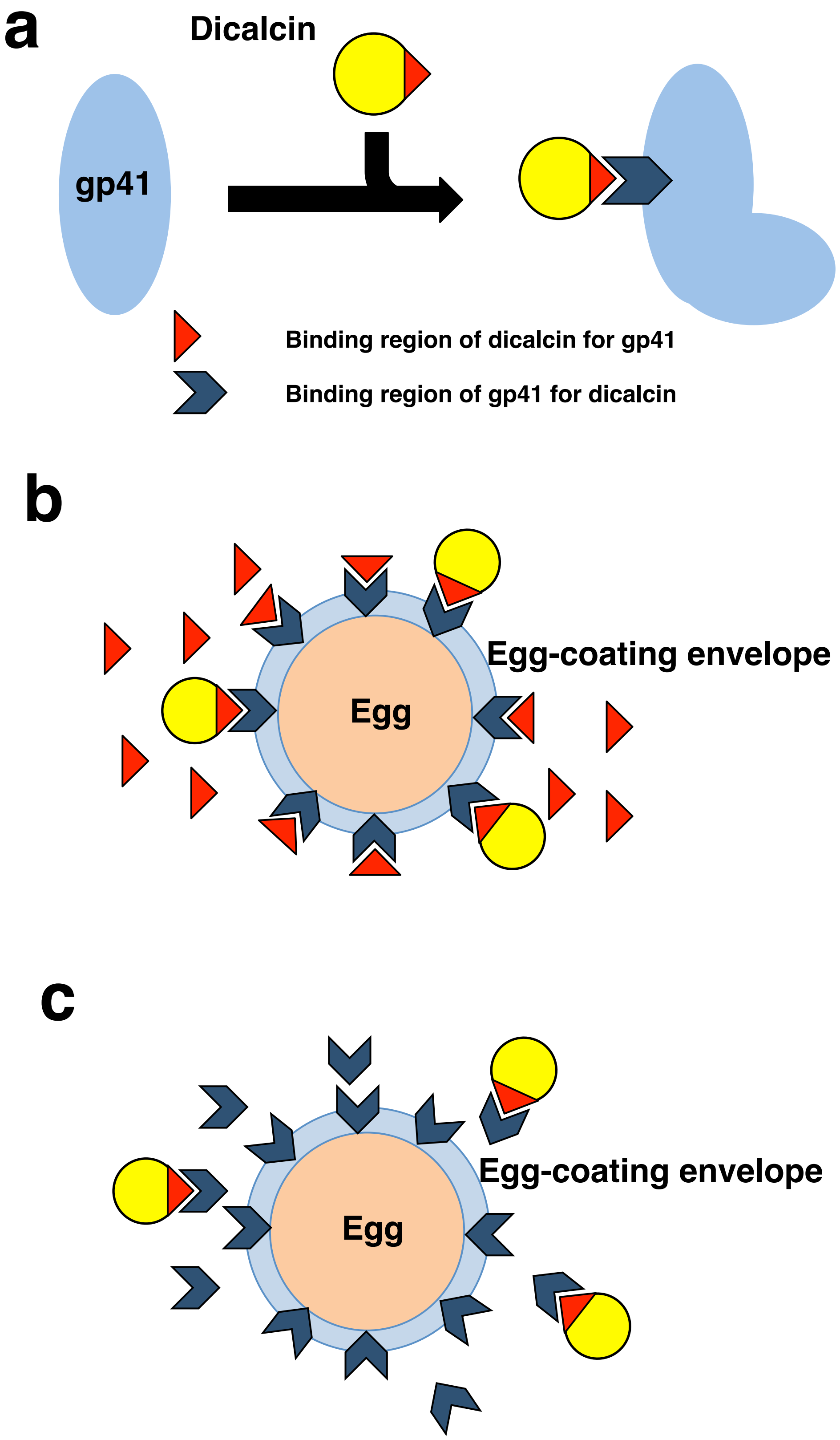

Supplementary Fig. S1

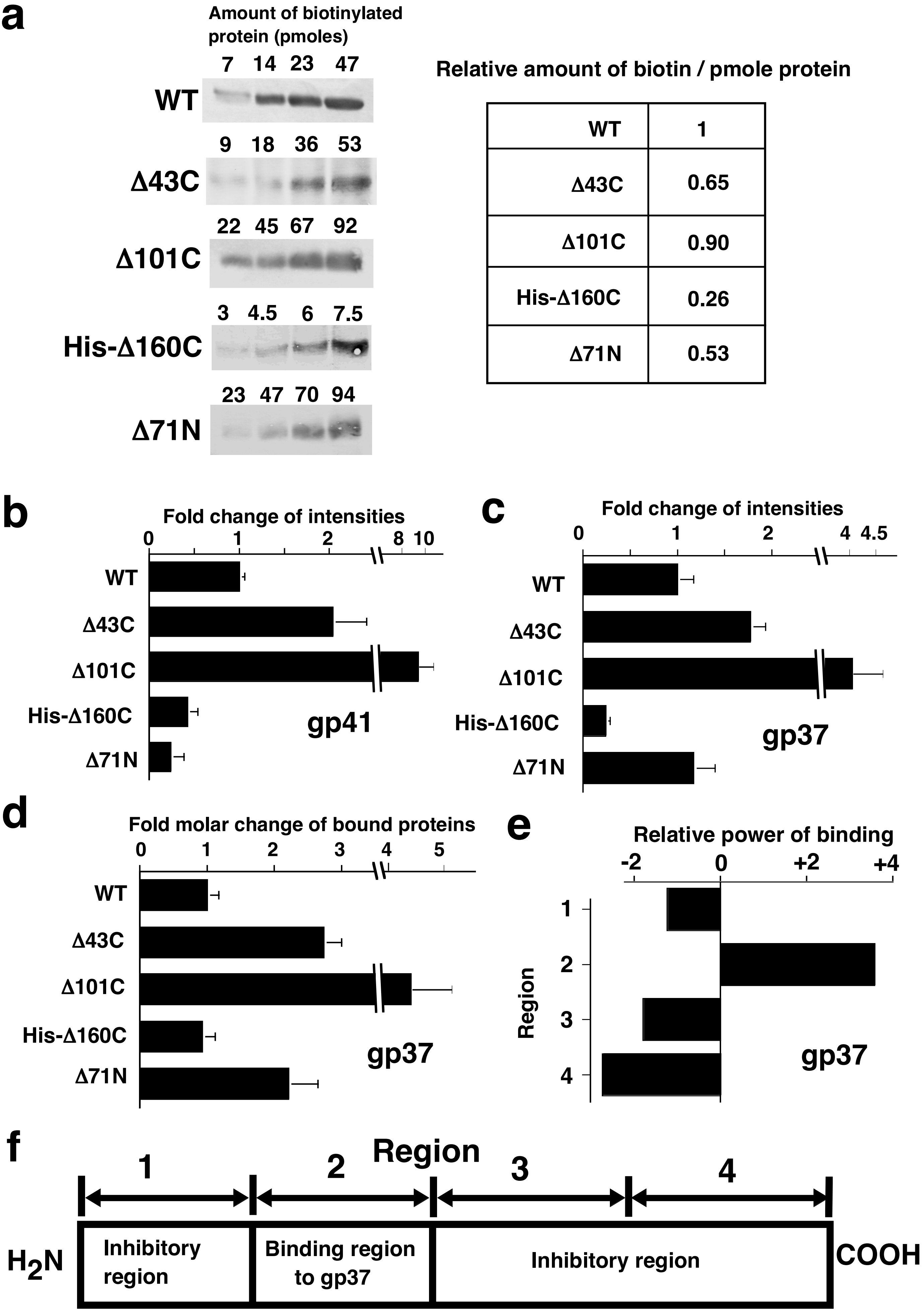

Supplementary Fig. S2

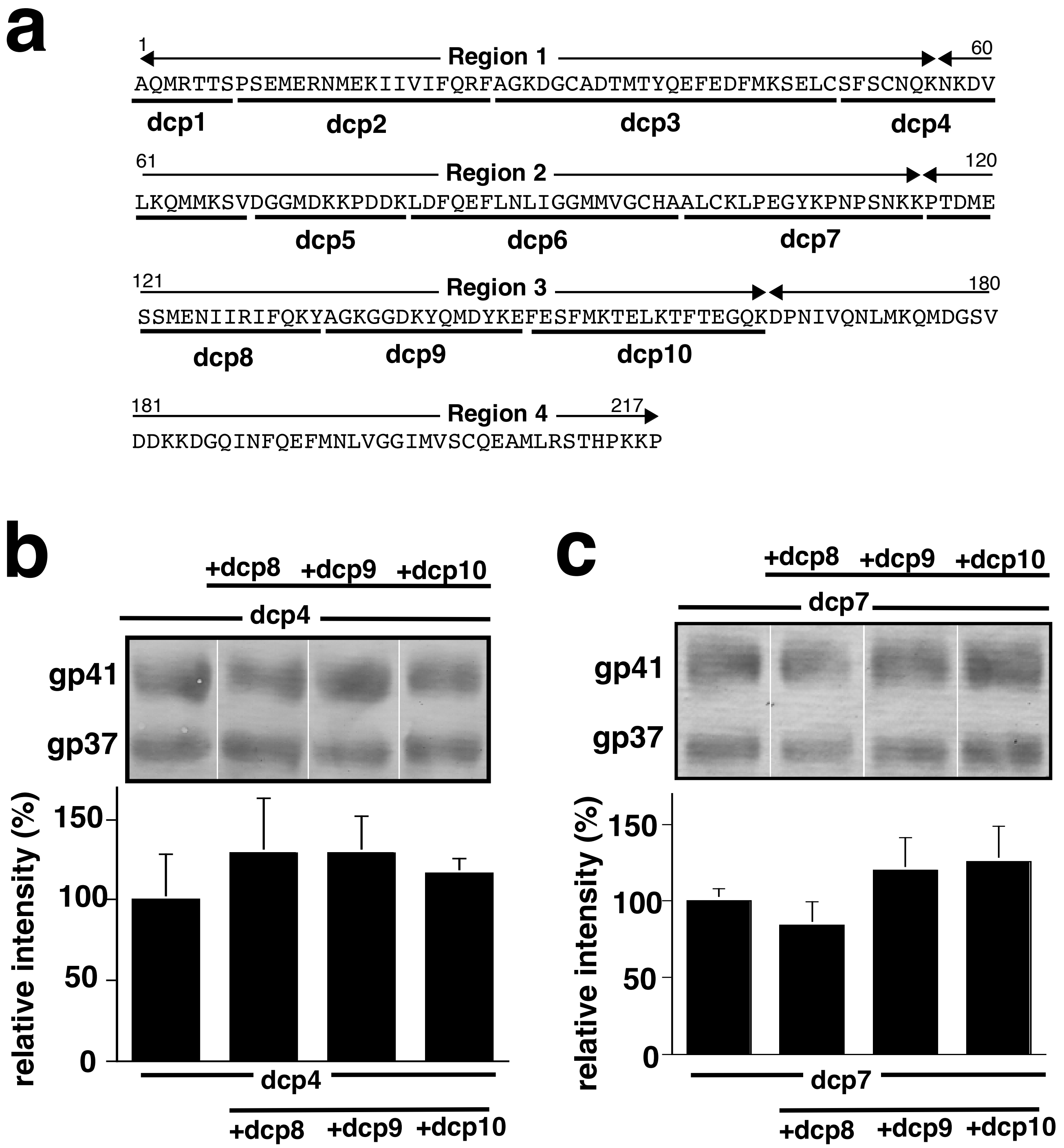

Supplementary Fig. S3

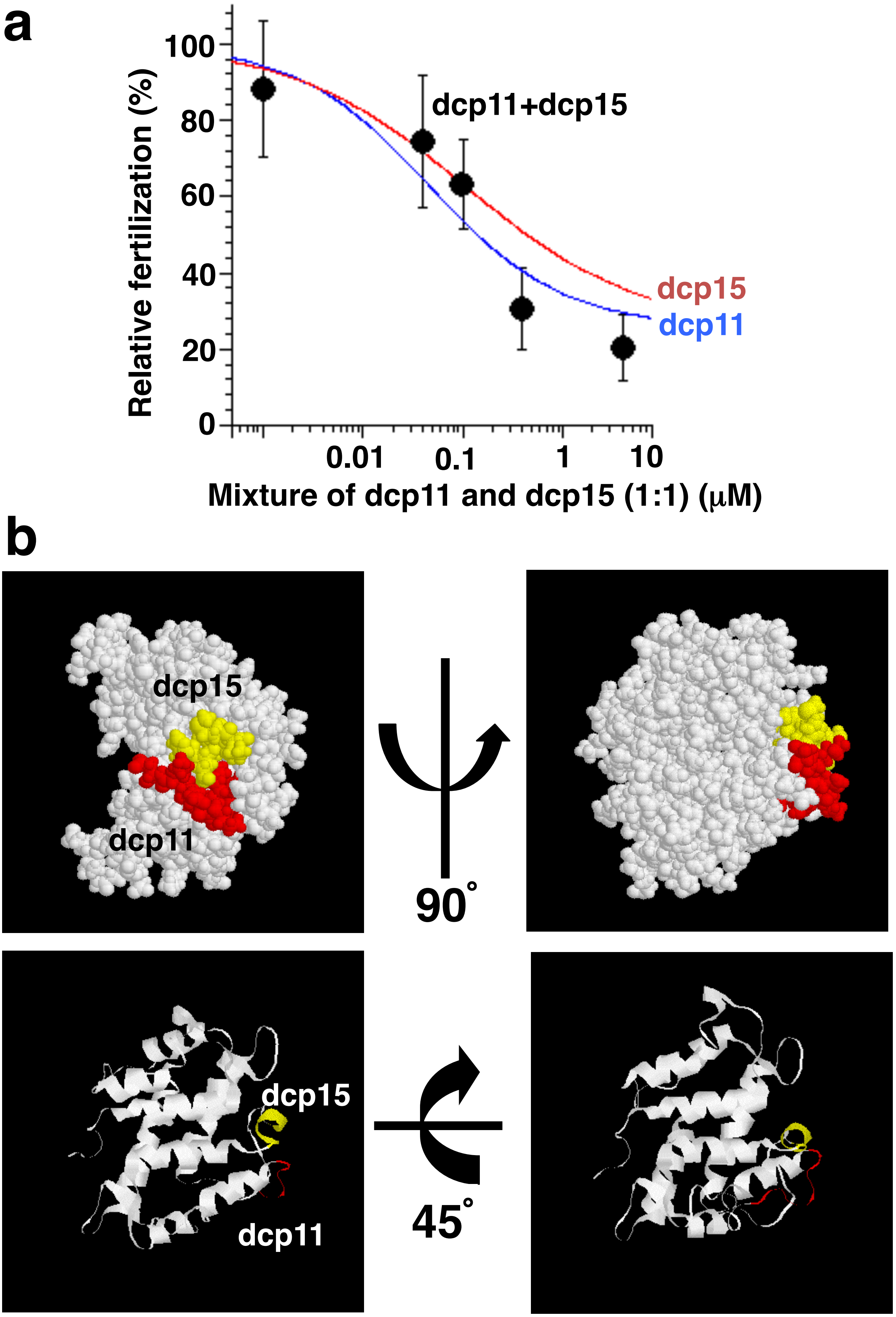

Supplementary FIg. S4

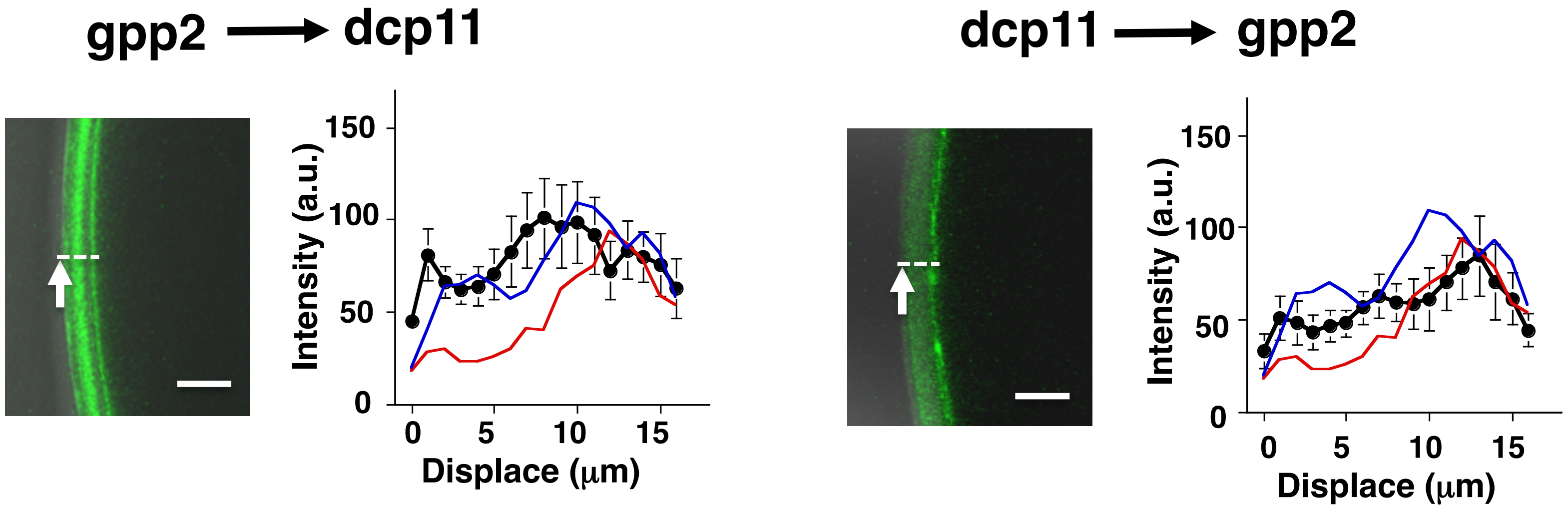

Supplementary Fig. S5
